# The Role of *α*-Synuclein and LRRK2 in Tau Phosphorylation

**DOI:** 10.1155/2015/734746

**Published:** 2015-04-21

**Authors:** Fumitaka Kawakami, Takafumi Ichikawa

**Affiliations:** Department of Regulation Biochemistry, Graduate School of Medical Sciences, Kitasato University, Sagamihara 2520373, Japan

## Abstract

There is now a considerable body of experimental evidence that Parkinson's disease arises through physiological interaction of causative molecules, leading to tau pathology. In this review, we discuss the physiological role of *α*-synuclein and LRRK2 in the abnormal phosphorylation of tau. In addition, as recent reports have indicated that heat shock proteins- (HSPs-) inducing drugs can help to ameliorate neurodegenerative diseases associated with tau pathology, we also discuss therapeutic strategies for PD focusing on inhibition of *α*-synuclein- and LRRK2-associated tau phosphorylation by HSPs.

## 1. Introduction

Parkinson's disease (PD), a chronic and progressive movement disorder caused by dopaminergic neuron loss in the substantia nigra, is the second most common neurodegenerative disease after Alzheimer's disease (AD). Although several causative genes of PD have been identified, the molecular mechanism responsible is not yet fully understood. The neuropathological hallmark of PD is the appearance of intraneuronal protein aggregates known as Lewy bodies (LBs) consistent with filamentous inclusions composed of *α*-synuclein (*α*-Syn). A histopathological study has detected the phosphorylated microtubule-associated protein tau in the LBs of some PD patients [1]. Abnormal phosphorylation of tau is known to be implicated in the pathogenesis of diverse neurodegenerative diseases, such as AD, PD, dementia with Lewy bodies (DLB), Pick's disease, progressive supranuclear palsy (PSP), frontotemporal dementia and parkinsonism linked to chromosome 17 (FTDP-17), and frontotemporal lobar degeneration (FTLD). Accordingly, neurodegenerative disorders characterized by aggregation of phosphorylated tau are termed tauopathies [2]. Although the underlying molecular mechanisms are still unclear, it is thought that tauopathies share common pathways of abnormal tau phosphorylation. The picture is a complex one because phosphorylated tau pathology occurs in different areas of the brain in each tauopathy, and different causal molecules are involved in the pathogenesis. We hypothesize that these disease-specific molecules may be able to induce abnormal phosphorylation of tau in a brain region-specific manner. Thus, identification of the upstream factors leading to abnormal tau phosphorylation would lead to a fuller understanding of the pathogenetic pathways associated with the tauopathies.

Tau is one of the microtubule-associated proteins that promotes tubulin assembly and stability of microtubule and is predominantly expressed in the central nervous system [3, 4]. Tau has six isoforms, ranging in size from 352 to 441 amino acid residues, which are produced by alternative mRNA splicing of the microtubule-associated protein tau (MAPT) gene [5–7]. In humans, the shortest isoform is expressed only in fetal brain, while all six isoforms are developmentally expressed in adult brain [8, 9]. Tau contains a proline-rich domain in its central region, consistent with seven P-X-X-P motifs, and its C-terminal region includes three or four repeated microtubule-binding domains [10–12]. It is well known that tau regulates neurite outgrowth and axonal transport by modulating the assembly of microtubules, a process critical for neuronal plasticity and memory consolidation [13]. The microtubule-binding ability of tau is regulated by several posttranslational modifications, including multiple phosphorylation, acetylation, glycosylation, ubiquitination, and sumoylation [14]. Among these, phosphorylation is thought to be a critical event in both normal regulation of tau function and the pathogenesis of tau-related neuronal degeneration. Tau contains a phosphorylation site of more than 80 Ser/Thr residues, and the process is catalyzed by several tau protein kinases, including glycogen synthase kinase 3 (GSK-3), cyclin-dependent kinase 5 (Cdk5), MAP-regulating kinase (MARK), c-Jun N-terminal kinase (JNK), protein kinase A (PKA), casein kinases 1 and 2 (CK1 and CK2), Ca^2+^/calmodulin-dependent protein kinase II (CaMKII), and protein kinase C (PKC) [15]. It has been revealed that highly phosphorylated tau forms a paired helical filament (PHF) [16, 17], and accumulation of this PHF in neurons and glial cells is one of the pathological hallmarks of AD. As a major tau kinase, GSK-3*β* phosphorylates numerous sites on tau, and site-specific phosphorylation of tau by this kinase significantly reduces its tubulin-binding ability, resulting in neuronal cell degeneration [18]. Therefore, extensive studies have focused on GSK-3*β* as a potential therapeutic target for neurodegenerative diseases associated with tau pathology.

There is now accumulated pathological and biochemical evidence for an association of *α*-Syn and leucine-rich repeat kinase-2 (LRRK2) with tau-related neuronal pathology. The presence of abnormally phosphorylated tau in LBs and the brain lesions of some PD patients with LRRK2 mutations strongly suggest that *α*-Syn and LRRK2 are closely associated with PD tau pathology. Our previous biochemical studies have indicated that *α*-Syn and LRRK2 interact directly with GSK-3*β* and enhance tau phosphorylation by the kinase [19–21]. Against this background, therefore, in the present review we discuss the molecular mechanisms of tau phosphorylation induced by *α*-Syn and LRRK2 as PD-related molecules and the possibility of developing therapeutic targets.

## 2. The Role of *α*-Synuclein in Tau Phosphorylation

*α*-Syn is a soluble protein composed of 140 amino acids and is abundantly detectable in the nucleus and presynapse of the neuronal cell [22]. *α*-Syn is considered to be involved in functions such as the regulation of neuronal vesicular transmission and synapse plasticity [23]. *α*-Syn comprises about 1% of total protein in the neuronal cytoplasm and is mainly expressed in the cerebral neocortex, hippocampus, substantia nigra, thalamus, and cerebellum [24]. It has also been demonstrated that *α*-Syn is differentially expressed in different brain regions [25]. In the thalamus, large amounts of *α*-Syn can be detected in both mitochondria and cytoplasm, whereas in the cerebral cortex and cerebellum it is abundant in cytoplasm but not in mitochondria [26, 27]. It has also been shown that *α*-Syn interacts with the phospholipid plasma membrane and Golgi apparatus [28, 29]. In the event of neurotransmitter release, *α*-Syn acts as molecular chaperone protein for formation of the soluble NSF attachment protein receptor (SNARE) protein complex involved in the fusion of synaptic vesicles with the cell membrane by assisting with the folding and refolding of synaptic proteins [30]. Therefore, it is thought that *α*-Syn has a particularly important role in synaptic activation.

In the substantia nigra of PD patients, aggregates of the acidophilic protein, LBs, are evident, and *α*-Syn is known to be a major protein component of LBs [31]. Accordingly, it has been suggested that defects in the intracellular systems for degradation of *α*-Syn, resulting in its excessive accumulation, may be responsible for the loss of dopaminergic neurons observed in PD. Braak et al. [32, 33] have proposed that, in the early stages of PD, *α*-Syn aggregation begins in the olfactory bulb, medulla oblongata, and gut and then gradually spreads to the midbrain, ultimately reaching the cerebral cortex. However, the molecular mechanism of *α*-Syn aggregation and spread is not yet fully understood. Interestingly, a recent study has revealed that intracellular accumulation of *α*-Syn in pathogenic brain regions is a common pathological feature of many neurological disorders [34]. Accordingly, neurodegenerative diseases characterized by accumulation of *α*-Syn, including PD, DLB, and multiple system atrophy, are collectively referred to as synucleinopathies [34]. The current picture suggests that neurodegenerative diseases characterized by accumulation of *α*-Syn share many common pathways and pathogenic mechanisms. Several studies focusing on the biological and physiological correlations between *α*-Syn and tau have noted that (i) polymorphisms of *α*-Syn and tau are commonly associated with sporadic PD [35, 36] and that (ii) *α*-Syn and tau are colocalized in the same proteinaceous aggregates in some synucleinopathies and tauopathies, such as PD with dementia (PDD), DLB, and Lewy body variant of AD (LBVAD) [1, 37, 38].

In general, it has been considered that formation of LBs containing *α*-Syn and aggregation of abnormal phosphorylated tau are independent mechanisms that occur in different neurodegenerative diseases. However, a previous study has demonstrated that *α*-Syn and abnormal phosphorylated tau were colocalized in LBs in the brain tissue of patients with sporadic PD and DLB [1]. More detailed studies have shown that (i) *α*-Syn interacts directly with tau [39], (ii) the PD-linked neurotoxin 1-methyl-4-phenylpyridinium ion (MPP^+^)/1-methyl-4-phenyl-1,2,3,6-tetrahydropyridine (MPTP) increases the expression of *α*-Syn and the phosphorylation level of tau at Ser262, Ser396, and Ser404 in cultured neuronal cells and wild-type mice, but not in *α*-Syn-knockout mice [40], and (iii) PKA and GSK-3*β* are the kinases responsible for *α*-Syn-dependent phosphorylation of tau [41, 42]. In our recent biochemical study to clarify the molecular mechanisms of *α*-Syn-dependent phosphorylation of tau protein, we demonstrated that *α*-Syn directly stimulates the GSK-3*β*-mediated phosphorylation of tau at Ser396 and autophosphorylation of the kinase, without affecting the native kinase activity (phosphorylation of glycogen synthase) [21]. Another group has also demonstrated that phosphorylation of GSK-3*β* at Tyr216 in cultured cells is increased by MPP^+^ treatment [42]. It has been reported that intrinsic kinase activity of GSK-3*β* is enhanced by phosphorylation at Tyr216 catalyzed by certain Tyr-kinases and autophosphorylation of GSK-3*β* [43]. Although *α*-Syn does not induce Tyr216 phosphorylation of GSK-3*β*, it stimulates autophosphorylation of both the Ser and Thr sites* in vitro* [21]. As it has been suggested that activation of GSK-3*β* alone is insufficient to induce hyperphosphorylation of tau, GSK-3*β*-related phospho-tau pathogenesis may require certain mediators that can coassociate with GSK-3*β* and tau molecules. Our previous study showed that *α*-Syn bound directly to GSK-3*β* and that *α*-Syn formed a tripartite complex with tau and GSK-3*β in vitro* [21]. Taken together, these biochemical findings suggest that *α*-Syn-mediated formation of a tripartite complex with tau and GSK-3*β* initiates the phosphorylation of tau by GSK-3*β*.

The synuclein family comprises three different isoforms [22]. Although our previous study demonstrated that the *α*- and *β*-Syn isoforms equivalently stimulate GSK-3*β*-mediated phosphorylation of tau, the *γ*-Syn isoform was found to have no stimulatory effects on tau phosphorylation by GSK-3*β* [21]. The common participation of the *α*- and *β*-Syn isoforms in tau phosphorylation by GSK-3*β* may be attributable to their structural features. *α*-Syn is an intrinsically unfolded protein with an N-terminal domain (residues 1–60) comprising seven imperfect KTKEGV sequence repeats, a middle hydrophobic domain (residues 61–95) termed the non-Abeta component (NAC) domain, and a C-terminal domain with a large proportion of acidic residues (residues 96–140) [22]. Among the three Syn isoforms, the amino acid sequences of the N-terminal amphipathic regions and the hydrophobic NAC domain are well conserved. However, the C-terminal region of *γ*-Syn does not match those of the other two isoforms. Although tau binds only to the acidic C-terminal domain of *α*-Syn, GSK-3*β* binds to the N-terminal region containing both the KTEGV repeat and the NAC domain [21]. Thus, *γ*-Syn may not be able to form tripartite complexes with tau and GSK-3*β*, perhaps accounting for its lack of any stimulatory effect on tau phosphorylation by GSK-3*β*.

Previously, it was revealed that *α*-Syn and *β*-Syn exhibit similar levels of expression, although *α*-Syn mRNA decreases and *β*-Syn mRNA increases in an age-dependent manner [44, 45]. On the other hand, a comparative study has demonstrated an increase in the level of *α*-Syn mRNA and a decrease in the level of *β*-Syn mRNA in the substantia nigra of patients with PD, DLBD, and LBVAD [25]. Moreover, it has been reported that *β*-Syn is undetectable in LB, whereas the sequence homology and structure of *β*-Syn are similar to those of *α*-Syn [46]. From all of these observations, it has been suggested that *α*-Syn- or *β*-Syn-mediated hyperphosphorylation of tau may occur in different neurodegenerative diseases. However, the specificity and physiopathological significance of *α*- and *β*-Syn for hyperphosphorylation of tau remain to be elucidated.

It seems likely that *α*-Syn protein expression is upregulated by oxidative stress and mitochondrial dysfunction [42]. Under physiological conditions, the concentration of *α*-Syn in neurons is estimated to be 70–140 *μ*M [47], whereas the normal concentration of tau is estimated to be approximately 2 *μ*M [48, 49]. Thus, under normal conditions, the molar ratio of tau to *α*-Syn is 1 : 35–1 : 70. Our previous study has indicated that the optimum molar ratio of *α*-Syn and tau for inducing maximum phosphorylation by GSK-3*β* is 1 : 20 [21]. Thus, *α*-Syn-mediated hyperphosphorylation of tau by GSK-3*β* may occur only when *α*-Syn is present at high concentrations induced by cellular stresses, such as oxidative stress and mitochondrial dysfunction.

**Figure 1:**
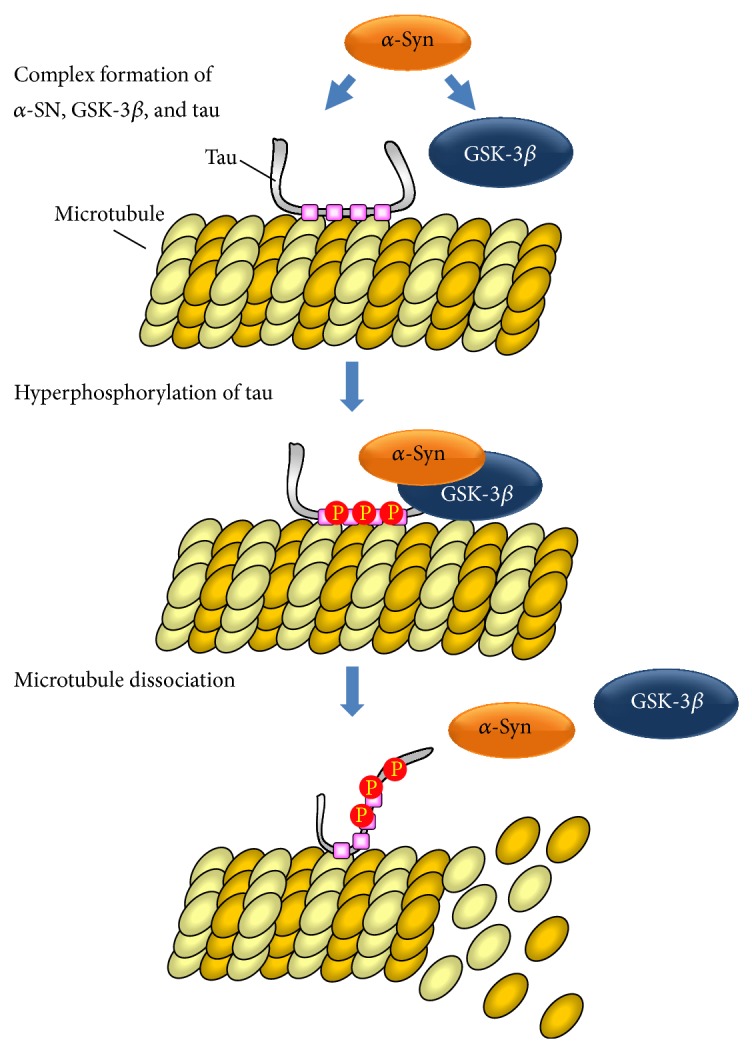
*α*-synuclein-mediated phosphorylation of tau.

In conclusion, activation of the intrinsic kinase activity of GSK-3*β*, defined by its Tyr216 phosphorylation, is insufficient to induce the hyperphosphorylation of tau. In our review, we hypothesize that a scaffolding molecule, such as *α*-Syn, capable of forming a complex between tau and GSK-3*β*, is absolutely essential for tau hyperphosphorylation by GSK-3*β* (Figure 1). Therefore, the development of molecules that can block the molecular interaction between *α*-Syn and GSK-3*β* is necessary for devising effective therapeutics for neurodegenerative diseases that result from tau hyperphosphorylation.

## 3. The Role of LRRK2 in Tau Phosphorylation

LRRK2 has been identified as the causal molecule of autosomal-dominant PD [50, 51]. Among all causative genes, mutations in the LRRK2 gene are the most prevalent cause of PD [52, 53]. LRRK2 mutations account for up to 7% of familial PD cases and up to 3% of apparently sporadic cases of PD [54]. In most PD patients with LRRK2 mutation, the clinical and pathological manifestations are indistinguishable from typical sporadic PD [55]. However, recent clinicopathological studies have shown that LB pathology is not detectable in all patients with PD-associated LRRK2 mutation [56], and therefore it has been suggested that LRRK2 mutations may be associated with the nonspecific neuronal degeneration commonly observed in PD.

LRRK2 is a 2527 amino acid protein with a molecular mass of approximately 250 kDa and unique domain architecture. LRRK2 is predicted to possess multiple functional domains, such as the armadillo repeat (ARM), ankyrin repeat (ANK), leucine-rich repeat (LRR), Ras of complex GTPase (Roc), the Roc C-terminal, a protein kinase, and WD40 (a tryptophan-aspartic acid repeat domain) [57]. Basic biochemical studies have demonstrated that wild-type LRRK2 exhibits Ser/Thr protein kinase activity and also autophosphorylation activity [58]. The kinase activity of LRRK2 is enhanced by binding of GTP (guanosine triphosphate) but is exerted independently of GTP binding and intrinsic GTPase activity [59, 60]. Furthermore, the ROC domain in LRRK2 has been shown to contribute to homodimer formation, and this dimerization enhances the kinase activity of LRRK2 [61–63]. Studies of protein localization have revealed that LRRK2 is widely expressed in many organs [50, 51]. In the normal brain, LRRK2 is distributed in the cerebral cortex, medulla, cerebellum, spinal cord, putamen, and substantia nigra [50, 64]. Accumulating pathological and physiological data indicate that the kinase activity of LRRK2 is potently enhanced by the G2019S mutation, which is the most common mutation of LRRK2 linked to the pathogenesis of PD [65]. However, it remains unclear whether other PD-associated mutations affect LRRK2 kinase activity. A number of previous reports have indicated candidate proteins that could act as substrates for phosphorylation by LRRK2, such as ezrin/radixin/moesin, eukaryotic translation initiation factor 4E-binding protein (4E-BP), *β*-tubulin, Akt, mitogen-activated protein kinase (MAPK), and MAP Kinase Kinases (MKK) 4 and 7 [66], although a robust physiological substrate for LRRK2 has not yet been defined. Nevertheless, many recent studies have revealed that the kinase activity of LRRK2 is critical for development of neurodegeneration and that LRRK2-mediated neuronal toxicity can be reduced by inhibiting the kinase activity both* in vitro* and* in vivo* [67]. Thus it seems that studies of the physiological function of LRRK2 focusing on its true substrates make it possible to understand the molecular mechanisms of neurodegeneration caused by LRRK2 mutations.

Our previous study has demonstrated that LRRK2 directly phosphorylates tubulin-associated tau but does not phosphorylate free tau [20]. Furthermore, in an* in vitro* experiment using recombinant tau and LRRK2, we identified Thr181 of tau as the direct target site for LRRK2 phosphorylation [20]. Another study using cultured cells has demonstrated that phosphorylation of tau at Thr181 and Thr231 is potently enhanced by LRRK2 [68]. More recently, Bailey et al. have reported that LRRK2 preferentially phosphorylates Thr149 and Thr153 [69]. All of these findings strongly suggest that tau acts as substrate for phosphorylation by LRRK2.

In the G2019S-LRRK2 transgenic mouse brain, an increased level of tau phosphorylation at Ser396/404 has been observed [70]. Since phosphorylation of tau at Ser396 is preferentially targeted by GSK-3*β* [18], we expected that LRRK2 might regulate this process through GSK-3*β*-dependent mechanisms. Therefore, we performed a similar experiment with *α*-Syn to determine the physiological relationship between LRRK2 and GSK-3*β* in the tau phosphorylation mechanism. We found that LRRK2 directly interacts with GSK-3*β*, and this interaction enhances the kinase activity of GSK-3*β in vitro* [19]. However, we found that there was no requirement for LRRK2 kinase activity in the activation of GSK-3*β* by LRRK2. From these results, it is speculated that upon interaction with LRRK2, GSK-3*β* undergoes a conformational change, thus activating its intrinsic kinase activity. We also demonstrated that phosphorylation of tau at Ser396 was increased in LRRK2-overexpressing SH-SY5Y cells. Thus, our study suggests that LRRK2 may also contribute to tau phosphorylation through a GSK-3*β*-dependent mechanism [19]. However, further detailed studies will be required to clarify the mechanism of LRRK2-mediated activation of GSK-3*β*.

Several previous studies have reported tau-related neuronal pathology in the brains of PD patients with LRRK2 mutations and also in LRRK2-transgenic animal models [69–73]. In particular, these reports indicated that (i) patients with G2019S mutation of LRRK2 have neurofibrillary tangles composed of phosphorylated tau as an atypical form of tau pathology [71], (ii) phosphorylation of tau is increased in R1441G- and G2019S-LRRK2 transgenic mice [70, 72], whereas tau phosphorylation is decreased in LRRK2-knockout mice [74], and (iii) tau-positive lesions are evident in patients with LRRK2-I2020T mutation [68]. Thus, LRRK2 may play an important role in tau phosphorylation-related neuronal pathology. However, the basic question that how PD-associated mutations of LRRK2 contribute to tau pathology is still unresolved. Relatively common PD-associated LRRK2 mutations have been found in or near the enzymatic domain of the protein. Among them, R1441C, R1441G, and R1441H exist within the ROC domain, and G2019S and I2020T mutations are located in the kinase domain. The G2019S-LRRK2 mutation unequivocally increases the kinase activity, but the effects of other pathogenetic mutations on the kinase activity have been controversial. In our previous study, the PD-associated LRRK2 mutations G2019S and I2020T were found to result in tau hyperphosphorylation* in vitro*, whereas the R1441C mutation did not [20]. We also found that G2019S-LRRK2 had higher binding affinity for GSK-3*β* than the WT or the other two mutants and that only the G2019S mutant enhanced GSK-3*β*-dependent tau phosphorylation. Thus G2019S-LRRK2 may contribute to abnormal tau phosphorylation through both direct and GSK-3*β*-dependent mechanisms. This hypothesis is strongly supported by a previous report describing that (i) G2019S-LRRK2-transgenic* Drosophila* neurons exhibit GSK-3*β*-mediated hyperphosphorylation and mislocalization of tau [75], (ii) G2019S-LRRK2 can bind strongly to GSK-3*β*, whereas R1441C does not bind to GSK-3*β* in the* Drosophila* model [75], and (iii) the ability of LRRK2 to bind to GSK-3*β* is specifically enhanced by G2019S mutation* in vitro*, whereas other mutations such as R1441C and I2020T have no such effect [19]. In another of our previous studies, we have shown that LRRK2 can interact with and phosphorylate Akt and that PD-associated LRRK2 mutants (R1441C, G2019S, and I2020T) show a reduced degree of interaction with and phosphorylation activity toward Akt in comparison with WT-LRRK2 [76]. The G2019S mutation, in particular, potently reduced the ability of LRRK2 to bind to Akt [76]. These results suggest that PD-associated mutations of LRRK2 may affect its ability to bind with downstream interacting partners. Interestingly, recent studies of Asian populations have shown that the LRRK2 gene variant G2385R is more frequent in PD patients than in normal individuals [77–80]. The G2385R mutation is located in the C-terminal WD40 domain of the LRRK2 molecule and is a risk factor for PD. Generally, it is thought that the WD40 domain contributes to protein-protein interaction, and therefore, C-terminal WD40 domain in LRRK2 may play an important role in the formation of complexes between LRRK2 and certain interacting proteins. Taken together, it is suggested that G2385R mutation may alter the selectivity of LRRK2 binding to its interacting partners. Thus, the G2385R variant may contribute to phosphorylated tau pathology via a specific pathway that differs from other LRRK2 variants in which the PD-associated mutation is located in the enzymatic region of the protein. Since tau phosphorylation is targeted by several tau protein kinases and not only by GSK-3*β*, LRRK2 mutants other than G2019S may contribute to tau phosphorylation through other tau kinase-dependent mechanisms. In fact, LRRK2 has been reported to interact with and phosphorylate Ste20 serine/threonine kinase family members (TAOK3, STK3, STK24, and STK25), TAOK3 being a kinase with high sequence homology to MARKK [81]. Therefore, it has been proposed that LRRK2 may enhance tau phosphorylation via the MARK cascade.

**Figure 2:**
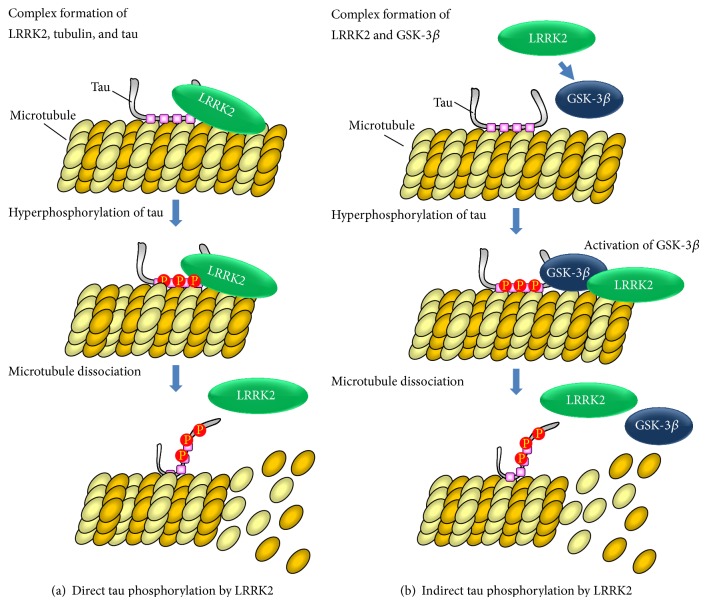
LRRK2-mediated phosphorylation of tau.

We conclude that LRRK2 contributes to tau pathology through abnormal tau phosphorylation resulting from both direct and indirect mechanisms (Figure 2). In future studies, it will be necessary to identify the specific partners interacting with LRRK2 variants and the individual downstream pathways in order to understand the molecular mechanisms of abnormal tau phosphorylation associated with LRRK2. If a specific blocker of LRRK2 binding to interacting molecules could be identified, it might show promise as a drug for treatment of neurodegeneration associated with abnormal tau phosphorylation in PD.

## 4. Therapeutic Strategy for PD Based on Inhibition of *α*-Syn- and LRRK2-Associated Tau Phosphorylation by Heat Shock Proteins

Heat shock proteins (HSPs) are a group of proteins that act as molecular chaperones and are involved in the folding and stabilization of numerous signaling proteins. They have cytoprotective functions against many forms of cellular stress, such as heat shock and oxidative stress [82]. In this section, we discuss the physiological relevance of HSPs to *α*-Syn and LRRK2, and we also propose the possibility of HSPs as therapeutic target for PD pathology based on the inhibition of tau phosphorylation by *α*-Syn and LRRK2.

Several studies have shown that HSP70 binds to the NAC domain of *α*-Syn and prevents aggregation of the latter [83] and also interacts with tau, inducing proteasomal degradation of the latter [84]. Our previous* in vitro* study has also revealed that HSP70 inhibits the *α*-Syn-dependent phosphorylation of tau by GSK-3*β*, without affecting the *α*-Syn-independent normal phosphorylation of tau by the kinase [21]. Furthermore, HSP70 prevents *α*-Syn-mediated complex formation between tau and GSK-3*β* by binding directly to the NAC domain of *α*-Syn [21]. These findings suggest that HSP70 may function as a physiological suppressor of *α*-Syn-mediated abnormal tau phosphorylation, without any toxic effect on the normal function of tau regulated by GSK-3*β*-mediated phosphorylation. In our previous study, we found that HSP70 suppresses the phosphorylation of tau by GSK-3*β* both* in vitro* [21] and in *α*-Syn-overexpressing cultured neuronal cells (unpublished data). Therefore, we consider that an HSP70-inducing agent might be effective for treatment of PD by preventing *α*-Syn-associated tau phosphorylation.

HSP90 interacts with LRRK2, and inhibition of this association leads to proteasomal degradation of LRRK2, but not via the lysosomal pathway [85, 86]. Furthermore, it is noteworthy that an HSP90 inhibitor can significantly reduce the protein expression of WT-LRRK2 as well as G2019S-LRRK2 in primary cortical neurons derived from G2019S-transgenic mice and transiently transfected HEK293 cells [86]. Therefore, HSP90 may play a key role in maintaining the stability of mutant and WT-LRRK2 protein in living cells. Interestingly, this report also indicated that treatment of cultured cells with an HSP90 inhibitor promoted the interaction of LRRK2 with HSC70 [86], which is an isoform of the HSP70 family constitutively expressed in mammalian cells. On the basis of these findings, we propose that HSC70 acts as a physiological inhibitor of LRRK2-mediated tau phosphorylation by interfering with the binding of LRRK2 to GSK-3*β*, which is required for activation of the latter. In addition, HSC70 shows over 90% sequence homology with HSP70 [82]. HSP70 has been reported to prevent the aggregation of LRRK2 in cultured cells [83], suggesting that HSP70 can interact with LRRK2, and thus may also interfere with formation of the LRRK2/GSK-3*β* complex.

Previous pharmacological studies have shown that inhibition of GSK-3*β* reduces tau phosphorylation and aggregation in a mouse model [87]. Some pharmaceutical companies are now conducting clinical or preclinical trials of GSK-3*β* inhibitors with the aim of developing therapies for AD [88]. Up to now, a variety of effective small inhibitors for GSK-3*β* have been developed using pharmacological approaches, but details of their possible side effects have yet to emerge. GSK-3*β* was first discovered as a protein kinase involved in the physiological regulation of glycogen metabolism, and a number of subsequent biochemical studies demonstrated that it has diverse physiological functions in the cell [89]. Therefore, GSK-3*β* inhibitors may affect not only the abnormal signaling pathway implicated in neurodegeneration but also normal pathways required for maintaining physiological function. Indeed, it has been shown that lithium, a classical GSK-3*β* inhibitor, exerts toxic side effects in some elderly patients [88], and therefore such studies have now been discontinued. Based on our previous findings, it is expected that molecules interacting with *α*-Syn or LRRK2, which are capable of preventing the formation of *α*-Syn- or LRRK2-mediated tripartite complexes with tau and GSK-3*β*, may act as more specific inhibitors of abnormal tau phosphorylation, without affecting normal tau phosphorylation by GSK-3*β*. As described above, HSP interacts with *α*-Syn and LRRK2 [85, 90] and prevents their aggregation or accelerates their degradation. Therefore, HSP-inducing drugs may be useful therapeutic agents for PD based on inhibition of *α*-Syn- and LRRK2-associated tau phosphorylation catalyzed by GSK-3*β*.

Currently, the protective function of HSP70 against AD pathology has been highlighted by the fact that some HSP70-inducing agents such as estrogen, geranylgeranylacetone (GGA), curcumin, and celastrol affect AD pathology at different pathological levels [91]. In particular, GGA has been shown to exert nontoxic HSP-inducing activity in various cell types [92] and has already been approved as a safe antiulcer drug for treatment of gastrointestinal mucosal lesions in humans. Therefore, HSP70 inducers such as GGA may suppress abnormal tau phosphorylation associated with *α*-Syn and LRRK2. Geldanamycin, a classical HSP90 inhibitor, may suppress LRRK2-mediated tau phosphorylation by reducing the protein expression of LRRK2, because inhibition of HSP90 binding with LRRK2 leads to proteasomal degradation of LRRK2. In addition, this drug increases the expression of HSP70 and HSP40 via activation of the HSF-1 transcriptional factor [93]. Therefore, HSP90 inhibitors may also prevent *α*-Syn- and LRRK2-associated tau phosphorylation by inducing the expression of HSP70.

**Figure 3:**
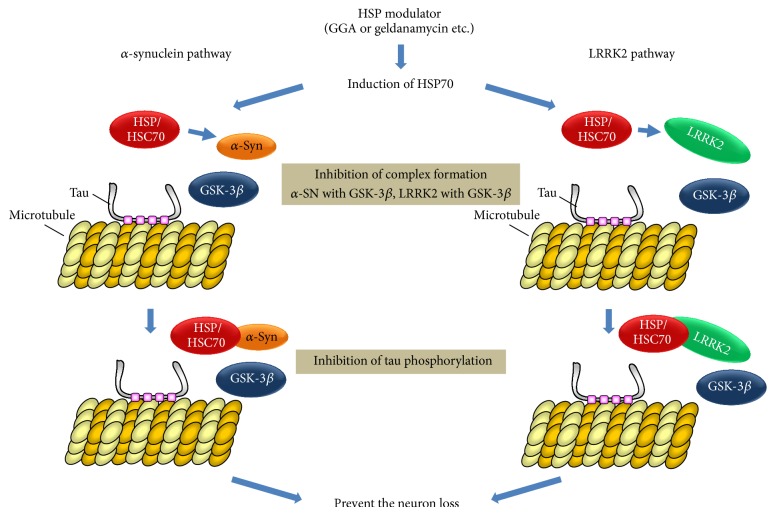
Suppression of Tau phosphorylation by HSP modulator.

Finally, we conclude that the pathogenetic mechanisms of tau phosphorylation involving *α*-Syn or LRRK2 might be prevented by HSPs. Therefore, we propose that induction of HSPs expression might be able to prevent the pathogenesis of PD associated with abnormal tau phosphorylation. As a future task, discovery of a pharmacological modulator of HSPs capable of crossing the blood-brain barrier would be important. This might be applicable as an effective and safe therapeutic drug for PD, preventing the abnormal phosphorylation of tau mediated by *α*-Syn and LRRK2 (Figure 3).
